# Characterizing redescriptions using persistent homology to isolate genetic pathways contributing to pathogenesis

**DOI:** 10.1186/s12918-015-0251-2

**Published:** 2016-01-11

**Authors:** Daniel E. Platt, Saugata Basu, Pierre A. Zalloua, Laxmi Parida

**Affiliations:** Computational Biology Center, IBM T. J. Watson Research Center, 1101 Kitchawan Rd., Yorktown Hgts, 10598 NY USA; Department of Mathematics, Purdue University, 150 N. University St., West Lafayette, 47907 IN USA; Graduate Studies and Research, Lebanese American University, P.O. Box 13-5053, Chouran Beirut, 1102 2801 Lebanon; Department of Environmental Health, Harvard University, 401 Park Drive, Boston, MA USA

**Keywords:** Pattern discovery, Redescription, Persistent homology, Genetic association, GWAS, Missing heritability

## Abstract

**Background:**

Complex diseases may have multiple pathways leading to disease. E.g. coronary artery disease evolves from arterial damage to their epithelial layers, but has multiple causal pathways. More challenging, those pathways are highly correlated within metabolic syndrome. The challenge is to identify specific clusters of phenotype characteristics (composite phenotypes) that may reflect these different etiologies. Further, GWAS seeking to identify SNPs satisfying multiple composite phenotype descriptions allows for lower false positive rates at lower *α* thresholds, allowing for the possibility of reducing false negatives. This may provide a window into the missing heritability problem.

**Methods:**

We identify significant phenotype patterns, and identify fuzzy redescriptions among those patterns using Jaccard distances. Further, we construct Vietoris-Rips complexes from the Jaccard distances and compute the persistent homology associated with those. The patterns comprising these topological features are identified as composite phenotpyes, whose genetic associations are explored with logistic regression applied to pathways and to GWAS.

**Results:**

We identified several phenotypes that tended to be dominated by metabolic syndrome descriptions, and which were distinct among the combinations of metabolic syndrome conditions. Among SNPs marking the RAAS complex, various SNPs associated specifically with different groups of composite phenotypes, as well as distinguishing between the composite phenotypes and simple phenotypes. Each of these showed different genetic associations, namely rs6693954, rs762551, rs1378942, and rs1133323. GWAS identified SNPs that associated with composite phenotypes included rs12365545, rs6847235, and rs701319. Eighteen GWAS identified SNPs appeared in combinations supported in composite combinations with greater power than for any individual phenotype.

**Conclusions:**

We do find systematic associations among metabolic syndrome variates that show distinctive genetic association profiles. Further, the systematic characterization involves composite phenotype descriptions that allow for combined power of individual phenotype GWAS tests, yielding more significance for lower individual thresholds, permitting the exploration of SNPs that would otherwise show as false negatives.

## Background

Coronary artery disease (CAD) is a multifactorial disease with inherited and behavioral components. Disease progression starts with any of a diverse set of injuries to arterial epithelial layers, which may take years to develop. These pathways lead to distinctive patterns of damage, e.g. with diabetes damage distinct in character from focal plaques associated with cholesterol. CAD risk factors cluster in highly correlated conditions called metabolic syndrome with distinct etiologies and pathways. Yet, the odds ratio associated with a pathway producing CAD is identical to the odds ratio that CAD was caused by that specific pathway, leading to diluted signals. Genome Wide Association Studies (GWASs) promised to reveal which Single Nucleotide Polymorphisms (SNPs) are clear causes of CAD and other diseases. Yet, identified SNPs only account for around 10 % of CAD, leaving 90 % of the heritability component unexplained [1–3]. One possible gap is the rather large possibility of false negatives given the high threshold excluding false positives in genome-wide surveys. Also, to isolate SNPs specific to a condition, genome-wide logistic regressions explicitly subtract the effects of other risk factors by including “adjustments”. Therefore, isolating genetic impact specific to CAD excludes the genetics of pathway-induced pathogenic etiology.

In this study, we sought associations connecting groups of phenotypic descriptions together against which we sought genome wide associations [4]. We identified significant phenotypic associations using pattern discovery to select combinations of factors appearing more or less often than expected by chance [5, 6]. Logistic regressions applied to such patterns then yield significant associations.

Pathway mechanisms create associations such that some phenotype condition *A* implies the presence of condition *B*, or *A*⇒*B*, and therefore also that *A*=*A*∧*B*. Therefore, the list of subjects *S*(*A*) associated with condition A will be the same as the list *S*(*A*∧*B*)=*S*(*A*)∩*S*(*B*). Therefore, the list of subjects *S*(*A*) associated with condition A will be the same as the list *S*(*A*∧*B*)=*S*(*A*)∩*S*(*B*). This implies two important points. First, it is important to identify patterns. Second, given patterns, we want to identify patterns that apply to the same sets of subjects, e.g. that satisfy *S*(*A*∧*B*)=*S*(*A*). Further, patterns with meaningful content are those for which *P*(*A*|*B*)≠*P*(*A*), which may be distinguished from chance by application of a statistical test, such as a binomial test of *P*(*A*∧*B*)≠*P*(*A*)*P*(*B*). If the sample space does not resolve these features with sufficient power to distinguish from random sampling variation, associations between *A*∧*B* and other patterns that might be revealed by clustering are not likely to be meaningful. It would be expected that any pattern that is significant will also reveal significant contributions in more detailed analyses such as logistic regression, or information-based tests to identify associations where Simpson’s Pardox may be present [7], or other tests to clarify the content of these composite patterns. Secondly, those associations are also characterized by uncertainty in sampling, misclassification errors, and variability in the physiological progression of disease. This implies that the equality must be interpreted in a sense that allows for statistical variation. Therefore, we sought clusters of patterns identified by the same sets of subjects measured by Jaccard distances within a threshold typical of the variability expected for odds ratios describing the patterns. Such clusters represent the same groups of subjects that can be identified by any of a number of different patterns, or “fuzzy redescriptions”, [8] reflecting underlying pathway-specific etiology. As such, the patterns each become multicomponent phenotypes suitable for a genome-wide *p*-value threshold =6.338×10^−7^.

In the search for SNPs that may be relevant to specific pathways, these compound statements of phenotypes provide two benefits. First, they offer greater specificity identifying subgroups of subjects that are distinct from other subgroups. It is important to emphesize that while these subgroups are distinct, they may share some subjects in common with other subgroups characterized by other groups of subjects. If these patterns identify pathways, then we should be able to identify greater power for some SNPs being identified with these compound phenotypes. Second, since these compound phenotypes, and each pattern in the cluster, provides multiple tests for each SNP, the chances that a GWAS SNP would emerge by random sampling variation for these multiple tests is greatly reduced. Essentially, the threshold is equivalent to the product of the thresholds *α* for each of the individual tests. This reduces the level of threshold required to exclude false positivies for each individual phenotype test, which implies that those candidate SNPs excluded as false negatives for a single phenotype GWAS test with Boneferroni correction have a chance to be reconsidered multiple times at lower thresholds.

The structure of these clusters was further explored using computational topological analysis [9], seeking to identify Vietoris-Rips complexes, where the patterns are vertices, the Jaccard distances provide the filtration, and the lifetimes of the generators of interest are within the Jaccard threshold, employing JavaPlex [10] to compute annotated persistent homologies. The generator complexes are also candidate phenotypes.

We applied logistic regression to SNPs drawn from the RAAS complex, as well as GWAS predicting these composite phenotypes. We also identified SNPs individually predicting all conditions comprising composite phenotypes, requiring the joint significance to be genome wide. This greatly increases sensitivity, reducing the threshold excluding false negatives, but at the cost of requiring significance of for multiple factors.

## Results and discussion

**Fig. 1:**
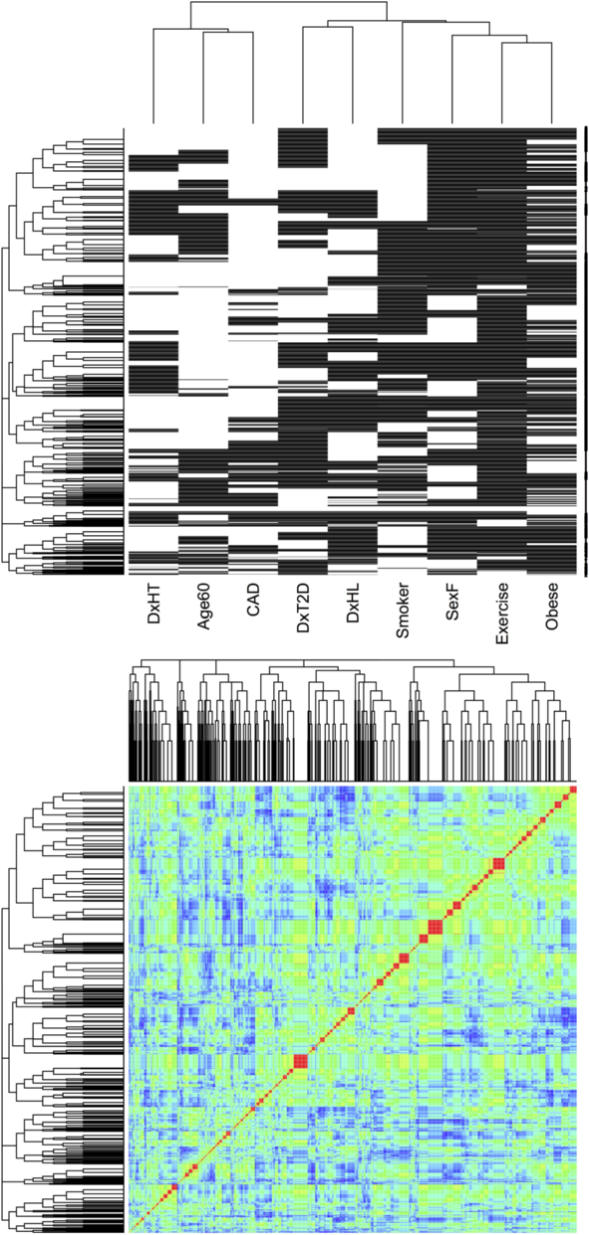
Two-way hierarchical clustering of binary threshold values of clinical variables with white positive, and distances plotted in section of rainbow from red to blue

Figure 1 shows a two-way biclustering of the binary coded thresholded clinical variables, and a rainbow coded plot (red smallest, blue largest) of Euclidean distances between enrollees based on the binary coded clinical values without scaling. Given the relatively strong association between clinical risk factors among metabolic syndrome patients, and that enrollees were all in a group for which an invasive coronary catheterization was deemed appropriate by physicians and therefore likely shared metabolic syndrome risk factors, it is notable that so many localized blocks of patients share features (larger red squares on the diagonal), but only moderate to low similarity connect blocks off-diagonal. The grey two-way cluster plot gives more of a sense of how the enrollees’ variables are organized within and around those blocks. This strongly suggests significant sub-organization within metabolic syndrome. Interestingly, there is not real evidence in this data set of an overall metabolic syndrome. Perhaps this is not surprising since all the subjects had been enrolled from a population of catheterized subjects, catheterization is an invasive procedure requiring adequate justification, and most subjects will have a history of metabolic syndrome as a part of their history justifying the catheterization.

**Fig. 2:**
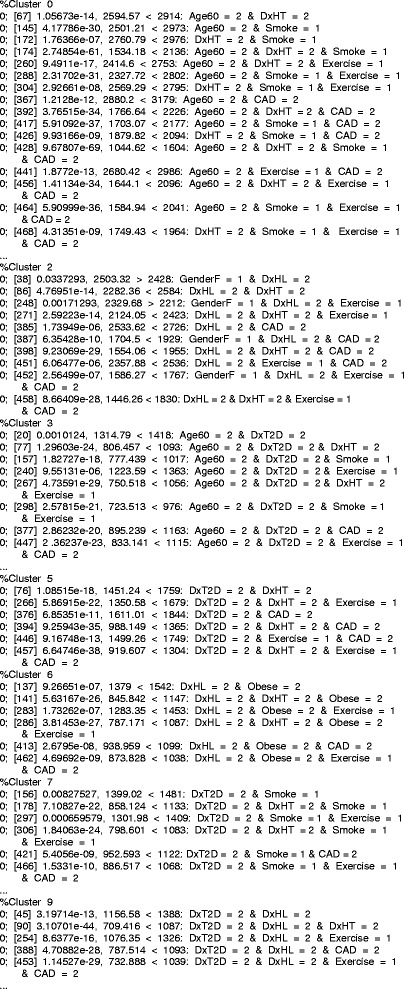
Patterns with redescription cluster identifications. First number is Fisher test for pattern list vs. cluster intersection. Second: pattern reference id. Third: binomial *p*-value. Fourth: expected count observed vs. observation marking khe tail of the binomial test evaluated. Last: list of columns and values. The Jaccard threshold was 0.25

**Fig. 3:**
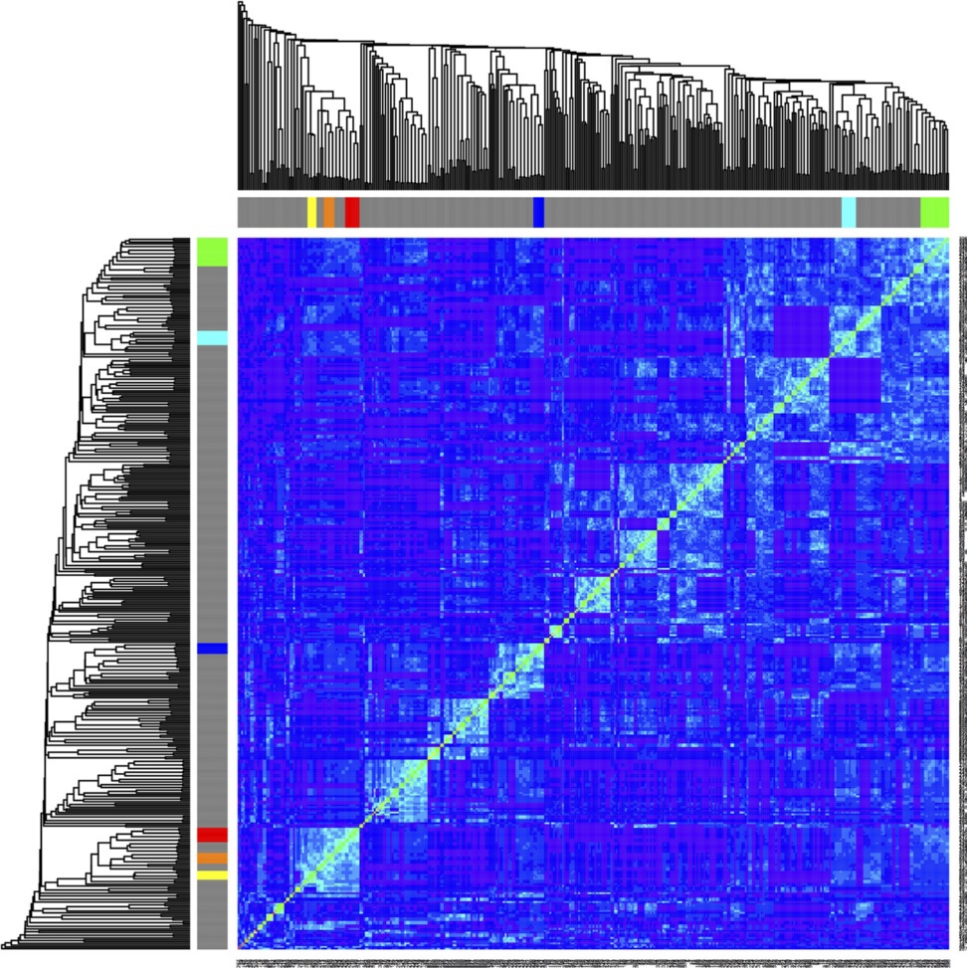
Distance clustering of the redescription Jaccard distances for significant patterns. This also serves as the filtration distance in the construction of the persistent homology analysis. Sidebar colors mark six redescription clusters. Red is Cluster 3 (Age ≥ 60 ∧ DxT2D ∧ DxCAD), Orange is Cluster 5 (DxT2D ∧ DxHT ∧ DxCAD), Yellow is Cluster 7 (DxT2D *wedge* non-smoker *wedge* DxCAD), Green is Cluster 0 (Age ≥ 60 ∧ DxHT ∧ DxCAD), Cyan is Cluster 2 (male ∧ DxHL ∧ DxCAD), and Blue is Cluster 6 (Obese ∧ DxHL ∧ DxHT ∧ DxCAD)

Logistic regression is a very common tool employed to assess the odds ratios of various risk factors in predicting disease, and especially for sorting out dependencies and interactions among risk variables in defining these associations. These interactions are graphically visible in the two-way heirarchical clustering map. This strongly suggests that a pattern discovery algorithm as described above may identify variable configurations likely to yield significant logistic regressions. Further, the relationships among patterns based on their enrollee sets may be used to recognize relationships among clinical variables describing the disease processes, and possibly pathways and stages. Figure 2 shows an example patterns, along with how the patterns cluster according to the Jaccard distances between the lists of enrollees matching the patterns. A total of 397 patterns were generated. Figure 3 shows the Jaccard distances displayed by heatmap organized according to single linkage hierarchical clustering. The sidebar colors mark the seven leading redescription clusters (nerves), at a Jaccard threshold of 0.30.

**Fig. 4:**
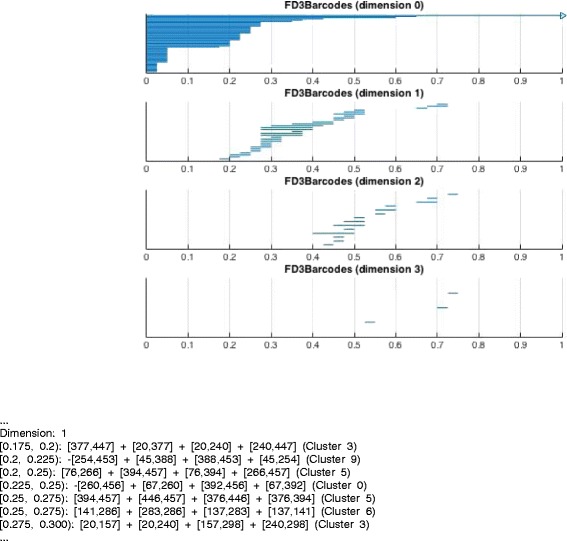
Persistent homology barcode plot of generators and an exerpt of the generator simplices from dimension 1

Figure 4 shows a barcode plot of generators, and an exerpt from the report of barcode correspondences indexed by the IDs of the patterns shown in Fig. 2. It is clear that the identified generators contained within the filtration of the nerve from Fig. 2 are subsets of the nerves. At larger filtration values, the barcodes tend to combine nerves. At those ranges, redescription clusters start to merge, with lower significance relating patterns to each other. The Jaccard distance measures the fraction of members of the two enrollee lists that are not shared between the patterns. So a distance exceeding 0.5 represents a situation where any given enrollee has less than fifty percent chance of being in both clusters, which is weak for inferring clinical relationships among patterns.

Table 1 shows prediction of complex phenotypes by RAAS complex and cytochrome gene SNPs. These include *NPPA* (rs5065), *REN* (rs6693954), *AGT* (rs699), *ADRB2* (rs1042713), *CYP1A2* (rs762551, rs1378942, rs1133323), and *ACE* (rs4343). The RAAS complex is a target of a number of common drugs targeting hypertension, such as angiotension-converting-enzime (ACE) inhibitors, angiotensin-receptor-blockers (ARBs), direct renin inhibitors target this complex. A number of SNPs show activity in a number of complex phenotypes including rs6693954, rs762551, rs1378942, and rs1133323. Of these, rs6693954 shows a slightly stronger odds ratio for the DxHT cluster than for DxHT, rs762551 and rs1378942 show stronger odds ratios for DxT2D and DxHT clusters than for their simple counterparts. While rs1133323 is significant for the DxT2D cluster, it is pretty clear that the cluster dilutes the simple DxT2D association. This is even more clear since the rest of the DxT2D cluster is identical to the DxHT cluster, and rs1133323 is not significant for DxHT or its cluster. This is somewhat striking in comparison to rs762551. The subcluster of patterns associated with the DxT2D cluster complex phenotype both identifies a number of different ways to identify the same set of enrollees, which suggests there is an underlying mechanism tying them together, and there are some SNPs that relate more positively to this cluster than just to the simple phenotype form. The relationship of this cluster to the DxHT cluster explored is also informative: they are distinct, and some SNPs more strongly associate with the complex DxHT phenotype than the simple phenotype.

**Table 1:** Odds ratio logistic regression associations of RAAS complex SNPs with RAAS and cytochrome P450 1A2

Locus	DxT2D	DxT2D	DxHT	DxHT	DxHL	DxHL
	cluster		cluster		cluster	
rs5065	0.93	0.94	1.01	0.99	0.96	0.96
G	(0.77–1.12)	(0.82–1.08)	(0.88–1.16)	(0.87–1.12)	(0.82–1.10)	(0.84–1.09)
	0.443	0.378	0.863	0.836	0.542	0.553
rs6693954	0.80	0.74	0.84	0.91	0.88	0.91
A	(0.66–0.98)	(0.63–0.85)	(0.72–0.99)	(0.79–1.05)	(0.76–1.03)	(0.79–1.06)
	0.029	6.22×10^−5^	0.0321	0.221	0.120	0.221
rs699	0.84	0.89	0.94	0.96	0.92	0.92
A	(0.67–1.06)	(0.75–1.06)	(0.79–1.13)	(0.81–1.13)	(0.77–1.10)	(0.78–1.09)
	0.138	0.182	0.541	0.632	0.366	0.333
rs1042713	0.93	0.94	0.99	1.01	0.98	1.03
A	(0.80–1.09)	(0.84–1.05)	(0.88–1.12)	(0.91–1.13)	(0.87–1.11)	(0.93–1.15)
	0.376	0.277	0.895	0.798	0.763	0.564
rs762551	1.42	1.23	1.28	1.17	0.93	0.94
C	(1.19–1.69)	(1.08–1.39)	(1.12–1.47)	(1.03–1.33)	(0.81–1.07)	(0.83–1.07)
	8.21×10^−5^	0.00203	0.000375	0.0188	0.297	0.332
rs1378942	1.33	1.19	1.27	1.22	0.97	0.99
C	(1.11–1.59)	(1.05–1.34)	(1.11–1.46)	(1.07–1.38)	(0.84–1.11)	(0.87–1.12)
	0.00154	0.00706	0.000672	0.00288	0.633	0.899
rs1133323	0.80	0.78	1.00	0.92	1.11	1.01
A	(0.66–0.97)	(0.68–0.90)	(0.86–1.16)	(0.80–1.06)	(0.96–1.30)	(0.88–1.16)
	0.0245	0.00758	0.983	0.235	0.164	0.884
rs4343	1.04	0.94	0.98	0.93	1.07	0.98
A	(0.90–1.20)	(0.87–1.09)	(0.88–1.10)	(0.84–1.04)	(0.95–1.20)	(0.88–1.09)
	0.618	0.646	0.785	0.200	0.273	0.698

Entries show locus w/ minor allele, and the odds ratio, 95 *p*-value for the SNP vs. the phenotype. DxT2D cluster = DxT2D ∧ Age ≥ 60 ∧ DxHT ∧ DxCAD, DxHT cluster = Age ≥ 60 ∧ DxHT ∧ DxCAD, DxHL cluster = DxHL ∧ Male ∧ DxCAD

SNPs rs6693954 and rs1133323 show a highly significant protective association against the simple T2D, but the compound phenotype shows a weaker association. Interestingly, rs6693954 had shown a non-significant HT association, but became significant when considering the compound phenotype. SNPs rs762551 and rs1378942 showed stronger associations for compound T2D and hypertension phenotypes than the simple phenotypes. These features indicate the compound phenotypes are resolving differences in how SNPs impact physiology in this population.

GWAS was applied to the dataset with composite phenotypes yielding the results in Table 2. It is interesting to note that, though rs12365545 (*MAML2*) appears in both DxT2D and DxHT results, it appears this may be dominated by association with DxCAD with Age ≥ 60. That association is a component of both, with OR = 1.42 (95 % CI 1.24−1.62), *p*-value =3.604×10^−7^. Yet, when predicting DxCAD by itself (no age threshold), the *p*-value is 0.00298. *MAML2* is mastermind-like 2 (Drosophila). It is implicated in several B cell-derived lymphomas, mucoepidermoid carcinomas, and chronic lymphocytic leukemia. It plays a role in regulation of ICN notch proteins, which have been implicated in T2D. rs6847235 (*GLRA3*) is intronic in glycine receptor alpha 3 which is a member of the ligand-gated ion channel protein family. rs701319 (intergenic) has no known clinical significance.

**Table 2:** Odds ratio logistic regression associations GWAS on compound phenotypes. Entries show locus w/ minor allele, and the odds ratio, 95 % confidence interval, and *p*-value for the SNP vs. the phenotype

Locus	Association	OR
Minor Allele		(95 % CI)
		*p*-value
rs12365545	DxT2D	1.69
A		(1.38–2.07)
		4.434×10^−7^
rs12365545	DxHT	1.44
A		(1.24–1.67)
		8.329×10^−7^
rs6847235	DxHL	1.45
A		(1.25–1.68)
		5.344×10^−7^
rs701319	DxHL	0.57
T		(0.46–0.71)
		6.821×10^−7^

Genome wide *p*-value threshold =6.338×10^−7^

Table 3 shows leading composite phenotypes. The “composite *p*-value” is the probability that uncorrelated random samples of null associations would have produced the pattern by chance. The “pattern *p*-value” is the actual logistic regression *p*-value for that SNP for the associated pattern. The SNP with the strongest pattern association in this group is rs12101936 (intergenic). rs17107637 (*SMOC1*) is intronic. *SMOC1* is *SPARC* related modular calcium binding 1, which appears to have a role in ocular and limb development. rs3759658 (*CGRRF1*) is in the cell growth regulator with ring finger domain 1 gene. rs6926556 (intergenic), rs8113086 (intergenic), and rs878643 (intergenic) show no known clinical association. rs3019548 (*CD6*) is possibly associated with multiple sclerosis. *CD6* encodes a protein found in the outer membrane of T-lymphcytes and some other immune cells, important for continuation of T cell activiation, contains three scavenger receptor cysteine-rich domains, and has a binding site for activated leukocyte cell adhesion molecule. rs6807700 (*PLSCR5*) is in phospholipid scramblase family, member 5, with no known clinical significance. rs 118800382 is in *ZNF331* (Taneera), which has been implicated in a T2D gene expression pathway analysis. rs2088354 is in the *SLC2a13* intron, a glucose transporter. rs2992100 is in MIR4500, a non-coding region. rs3781788 is in *MMP20*, a matrix metalloproteinase. rs6080252 is in *KIF16B*, coding a kinisen-like motor protein, involved with plus end motility of early endosomes and the balance between recycling and degredation of receptors, such as EGFR, FGFR. rs6984384 is in *LOC101929576* - an uncharacterized RNA gene. rs701133 is in *GPR149* - a G protein-coupled receptor. rs818710 is in *BSPRY*, B Box and *SPRY* Domain Containing Protein. rs17077265 and rs522264 are intergenic. rs6727857 is in *SCN2A*, Sodium Channel, Voltage-Gated, Type II, Alpha Subunit. All snps were significant, though not genome wide significant, except for rs8113086.

**Table 3:** SNPs present in each of the component phenotypes in compound sets, with composite *p*-value computed from individual phenotype components, pattern *p*-value computed on the compound phenotype members, odds ratio logistic regression associations GWAS for each phenotype set. Entries show locus w/ minor allele, and the odds ratio, 95 % confidence interval, and *p*-value for the SNP vs. the phenotype

Locus	Composite	Pattern	DxT2D	DxHT	DxCAD ∧
Minor Allele	*p*-value	*p*-value			Age ≥ 60
rs12101936	1.079×10^−9^	4.961×10^−5^	0.76	0.78	0.80
A			(0.64–0.89)	(0.67–0.90)	(0.69–0.93)
			0.000628	0.000615	0.002796
rs17107637	1.872×10^−8^	0.002181	1.41	1.37	1.29
G			(1.14–1.75)	(1.13–1.67)	(1.06–1.57)
			0.00135	0.00140	0.00992
rs3759658	8.914×10^−8^	0.0304	1.34	1.33	1.35
A			(1.08–1.66)	(1.09–1.61)	(1.11–1.65)
			0.00733	0.00455	0.00267
rs6926556	9.205×10^−9^	0.0341	0.74	0.72	0.76
C			(0.60–0.91)	(0.60–0.86)	(0.63–0.92)
			0.00438	0.000457	0.00460
rs8113086	2.853×10^−7^	0.0936	0.75	0.77	0.75
T			(0.60–0.93)	(0.64–0.93)	(0.62–0.92)
			0.00841	0.00760	0.00447
rs878643	4.254×10^−8^	0.04394	0.764	0.800	0.819
A			(0.65–0.90)	(0.69–0.93)	(0.70–0.96)
			0.00841	0.00760	0.00447
rs11880382	1.518×10^−7^	0.005167	0.733	0.901	0.889
C			(0.64–0.84)	(0.79–1.03)	(0.78–1.01)
			1.669×10^−5^	0.118	0.077
rs2088354	3.133×10^−8^	0.0144	1.679	1.357	1.175
T			(1.31–2.15)	(1.09–1.68)	(0.95–1.46)
			3.856×10^−5^	0.00557	0.146
rs2992100	4.162×10^−8^	0.06262	0.559	0.772	1.282
A			(0.43–0.73)	(0.60–0.99)	(1.00–1.64)
			2.35×10^−5^	0.0379	0.0468
rs3781788	4.837×10^−8^	0.001568	1.907	1.015	1.436
T			(1.44–2.52)	(0.77–1.34)	(1.09–1.89)
			5.527×10^−6^	0.913	0.00959
rs6080252	6.587×10^−8^	0.002838	1.623	1.303	1.279
A			(1.28–2.06)	(1.03–1.65)	(1.02–1.61)
			6.277×10^−5^	0.0284	0.0370
rs6984384	3.906×10^−8^	4.502×10^−5^	0.742	0.840	0.895
T			(0.65–0.86)	(0.74–0.96)	(0.79–1.02)
			4.886×10^−5^	0.008405	0.09511
rs701133	3.865×10^−7^	0.002081	1.547	1.206	1.160
T			(1.25–1.91)	(1.00–1.46)	(0.96–1.41)
			5.57×10^−5^	0.054	0.129
rs3019548	3.905×10^−7^	0.009792	1.541	1.483	–
G			(1.20–1.98)	(1.18–1.86)	
			0.000643	0.000607	
rs6807700	1.061×10^−7^	0.0002289	–	1.46	1.49
C				(1.18–1.80)	(1.20–1.85)
				0.000400	0.000265
rs17077265	2.80144×10^−7^	0.0131	–	1.409	1.062
T				(1.23–1.61)	(0.93–1.21)
				7.423×10^−7^	0.377
rs522264	2.53×10^−7^	2.238×10^−5^	–	0.638	0.734
T				(0.51–0.79)	(0.59–0.91)
				5.567×10^−5^	0.00455
rs6727857	4.819×10^−7^	0.000935	–	0.660	0.932
G				(0.56–0.78)	(0.79–1.10)
				1.173×10^−6^	0.411

Genome wide *p*-value threshold =6.338×10^−7^

The contrast between Tables 2 and 3 shows that standard GWAS, even treating compound phenotypes as simple phenotypes, picks out different SNPs than considering multiple tests applied by each of the compound phenotype components. This gives strong evidence that the compound approach yields SNPs that are false negatives that would otherwise be discarded as false positivies. While the *p*-values of some of the patterns (“pattern *p*-value”) are not as strong as might be suggested by the composite of individual phenotypes comprising the compound phenotype (“composite *p*-value”), there are a number of highly significant SNPs worth considering that standard methods miss.

## Conclusions

Associations between RAAS SNPs and metabolic syndrome characters, both clustered and singly, show specificity for conditions (e.g. rs1133323 for DxT2D clustered and unclustered, rs1378942 and rs762551 for DxT2D clustered and unclustered, and DxHT clustered and unclustered), and some specific to the complex phenotype (e.g. rs6693954 for DxHT clustered, but not DxHT) but not the single form, suggesting that the complex phenotypes convey more pathway specific information than individual simple phenotypes do by themselves.

GWAS applied to combined tests from the composite phenotype components shows significant lowering of the threshold for individual tests required to identify SNPs that are significant in multiple metabolic syndrome conditions, allowing for the possibility of relieving exclusion of some of the false negatives. The SNPs identified in individual tests are distinct from composite tests, indicating that different components of the sample space are probed by applying tests for each of the phenotypes represented in a composite pattern.

Several things may be explored in greater detail later. These include richer phenotype sets and more patterns. Another aspect is that the homology groups typically produced are sets of surfaces enclosing empty volumes, namely *H*_*n*_=ker(*∂*_*n*_)/Im(*∂*_*n*+1_), including the subset Im(*∂*_*n*+1_)/Im(*∂*_*n*+1_)≃{0}. These show the most topologically interesting features with conserved Betti numbers. However, other structures may be physiologically informative, such as *C*_*n*_/ker(*∂*_*n*_), which would represent forms that sprawl and do not enclose volumes.

## Materials and methods

### Datasets

Seven thousand six hundred thirteen subjects were selected from Lebanese patients enrolled as part of a multi center cross-sectional study for the FGENTCARD Consortium (http://www.well.ox.ac.uk/fgentcard/) [11] in a cross-sectional study. Patients were recruited from catheterized patients at the Rafic Hariri University Hospital and the Centre Hospitalier du Nord in Lebanon between May 2007 and June 2010. A questionnaire developed to measure the impact of CAD risk factors and family history was collected after informed consent was obtained from participants prior to conducting the study, as approved by the Lebanese American University institutional review board. Annotations were coded from medical charts for data such as laboratory tests, prescribed medications, and presence of other clinical conditions. Venous blood samples were drawn on EDTA. Of these, 6 were excluded due to missing information on age or sex, leaving 7607.

Nine hundred and ninety eight Lebanese Type II Diabetes (T2D) study participants were all of Lebanese origin and were recruited. A first recruitment campaign, conducted with the collaboration of the Lebanese University Medical Center, led to the recruitment of 506 subjects from the suburbs of Beirut, the capital of Lebanon. In a second campaign conducted in North Lebanon, 492 subjects were successfully recruited. Research was carried out in compliance with the Helsinki Declaration and with the approval of the LAU institutional review board and local ethics committees on human research (Reference number SMPZ08072010-4). All participants signed an informed consent and data and blood samples were obtained from each individual. By taking part in the two recruitment campaigns, participants (1) answered a detailed questionnaire, (2) gave a blood sample for DNA analysis and (3) gave a blood sample for HbA1C, fasting blood glucose (FBS), and lipid profile measures after 12 hours fasting. In this analysis, the T2D clinical data were not employed. The SNP data was used only to improve imputation analysis.

DNA was extracted using a standard phenol-chloroform extraction procedure. Two thousand seven hundred fifty two CAD study samples were analyzed on a number of platforms: 48 subjects’ DNA samples were analyzed using Human610-Quad beadchip and Illumina (582.775); 1055 subjects were analyzed using Human610-Quad beadchip and Illumina (582.892); 928 subjects were analyzed using Human Omni EXP – 12v1 multi-use; 706 with Illumina Human 660W Quad Beadchip; 7 with HumanOmniEXP-12v1 Multi-use + Human610-Quad Beadchip and Illumina (582.892); and 8 with HumanOmniEXP-12v1 Mullti-use + Illumina Hujan660W-Quad Beadchip. The 538 T2D DNA analyses were performed using Human Omni EXP – 12v1 multi-use. Seven hundred eighty nine thousand SNPs passed QC using PLINK 2 [12], (https://www.cog-genomics.org/plink2) for data management and quality control, keeping samples with call rate *ge* 95 %, SNPs call rate ≥ 90 %, MAF *ge* 1 %. BEAGLE ver 4.0 [13], (http://faculty.washington.edu/browning/beagle/beagle.html) was employed to impute SNPs among inconsistent chip SNP sets. SNPs with more than 2 alleles were removed.

Descriptors were coded for analysis as follows. DxT2D indicates diagnosis of type II diabetes. DxHT indicates diagnosis of hypertension. DxHL indicates a diagnosis of hyperlipidemia. Age60 marks age 60 years or older. SexF marks female. Obese indicates BMI levels in excess of 30. Smoker implies heavy cigarette smoking or hookah. Exercise marks regular intensive exericse. CAD marks greater than 70 % occlusion.

#### Demographics notes

Metabolic syndrome, CAD, and T2D prevalences are rapidly increasing in the Middle East, linked to recent changes in diet and activity in the population as a whole [14]. This rapid emergence argues for the possibility of age-structured changes in dietary habits and risk factors. There may be a delay of some years between earliest epithelial damage leading to CAD, or for progression from insulin resistance to full T2D. Further, emergence of CAD, hypertension (HTN), T2D and other metabolic syndrome conditions is strongly age and sex associated, while some risk behaviors are gender and age specific associated.

### Analysis

Metabolic syndrome is characterized by the strong association between obesity, hypertension, dyslipidemia, coronary artery disease, and type II diabetes mellitus. Many of these conditions emerge in older age. Among catheterized subjects, expectation would be that variables describing these conditions would cluster in well-defined patterns. To that end, hierarchical 2-way clustering was performed using R’s [15] heatmap function, invoking hclust() for clustering, and unscaled Euclidean distances between enrollees across clinical variables, and between clinical variables across enrollees. Also, heatmap was used to display unscaled Euclidean distances between enrollees mapped to a rainbow segment from red through blue.

The 2-way hierarchical clustering identifies blocks of relationships among descriptors shared among groups of subjects. The structure of those blocks may be explored in greater detail. Each descriptor *d* in the set of descriptors *D* has associated with it an alphabet *A*_*d*_ of values that *d* assumes among the set of subjects *S*. Each subject *sinS* has a descriptor tuple *q*(*s*)∈*Q*=×_*d*∈*D*_*A*_*d*_. The tupple member corresponding to *d*∈*D* is *q*_*d*_(*s*)∈*A*_*d*_ (pulldown). For each *a*∈*A*_*d*_, it is possible to identify the set of subjects $S{a} = q_{d}^{-1}(a) \subset S$ that “have” that value *a* of the descriptor.

The patterns *r*∈*R* are comprised tuples similar to the subject tuples except that the alphabet is augmented by a wild card "*" such that $q_{d}^{-1}(*) = S$. Then *R*⊂×_*d*∈*D*_(*A*_*d*_∪{∗}). Using the symbol *r*_*d*_ for the descriptor *d*∈*D* on *R* (pulldown), it is possible to identify *r*_*d*_(*r*)∈*A*_*d*_∪{∗}. Then it is possible to identify a set of subjects *S*(*r*) “matching” a pattern *r* as $S(r) = \bigcap _{d \in D} q_{d}^{-1}\left (r_{d}(r)\right)$ The total number of possible patterns is then $\prod _{d \in D} (\vert A(d) \vert + 1) - 1$, where the pattern comprised entirely of wild cards is excluded.

Blocks that appear in multiple descriptors horizontally across the plot in the two-way hierarchical clustering may be significant, and correspond to some *S*(*r*) for *r*∈*R*. It is expected that biological processes will tend to correlate descriptors among the sampled subjects. The difficulty is that such correlations may appear simply by chance. One way to exclude chance is to consider the fraction *p* of samples that would be expected given no correlation in some pattern *r*: $p(r) = \prod _{d\in D} \frac {\vert S{r_{d}} \vert }{\vert S \vert }$. Then the chances of finding more than |*S*_*q*_| by variation in random sampling would be binomially distributed with $P = \sum _{n \ge \vert S(r) \vert } \binom {\vert S \vert }{n} p(r)^{n} (1-p(r))^{\vert S\vert - n}$. Likewise, if the *r* are anticorrelated, then the probability of finding fewer by chance due to random sampling variation would be $P = \sum _{n \le \vert S(r) \vert } \binom {\vert S \vert }{n} p(r)^{n} (1-p(r))^{\vert S\vert - n}$.

**Fig. 5:**
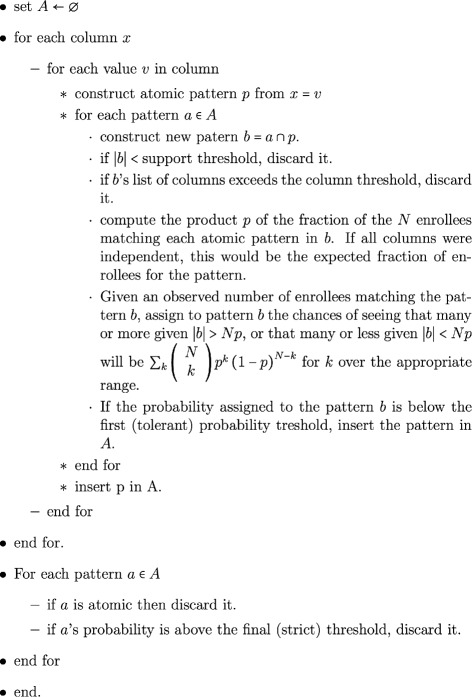
Algorithm used to generate patterns

Such patterns were identified using a pattern recognition algorithm defined in Fig. 5.

The use of a support threshold acknowledges that lower support patterns will tend to have lower statistical power. Discarding low support is an option that limits the number of weak patterns that are carried along in the computation. There are two significance levels. It is conceivable that some complex patterns may have a stronger significance level than some of the intermediate patterns. Allowing two support levels permits the retention of weaker patterns which are removed at the end with the stronger and final significance threshold.

If two patterns *A*=*A*∩*B*, then *A*⊆*B*, or *A*⇒*B*. Sets of patterns that yield equal sets of enrollees are called “redescriptions” [8]. Obviously, these redescriptions imply more complex relationships among patterns and possible underlying disease mechanisms and processes. Since thresholds may not be perfect, misclassification errors, disease progression changes, etc, tends to inject variability into the analysis. Association studies may show significant association and still allow for substantial amounts of variance. For this reason, Jaccard distances measured on the list of enrollees for each pattern are computed as dissimilarities that satisfy metric conditions. Assignment of nearest neighbors by Jaccard distance below some threshold yields pairwise connectivity, from which clusters are constructed using an algorithm resembling floodfill. These clusters are “fuzzy redescriptions”. Their utility emerges only if the threshold is low enough so that it would be unlikely for random processes to have constructed such clusters by chance. Fisher exact tests may be applied to assess whether random processes could have produced pairs by chance, and have been applied here between any pattern in the cluster against the intersection of all cluster members.

Rediscriptions also relate patterns to each other, some according to implications, or perhaps more generally in terms of shared physiology related to a disease process. Coronary artery disease is a “complex disease”, with multiple possible pathways leading to epithelial damage in arteries, and ultimatley to formation of plaques. These clusters of descriptions may pick out phenotypic characterization of underlying pathways at specific stages of pathogenic development.

Since we have a set of patterns with a well-defined metric distance between them, it is also possible to use the Jaccard distance as a filtration index in a persistent homology computation. In this context, the fuzzy redescription clusters defined above correspond to a “nerve”. One point worth noting is that the whole range of persistence is not necessarily interesting; once distances are long enough that the chances that clusters formed by chance is relatively high, there is really no information from which relationships between patterns (e.g. corresponding to *A*=*A*∩*B*) may be derived.

Within the persistent homologies, the generators carry information about topological structures within the nerves that may reveal yet more detail about pathological development specific to disease pathways.

Given that each pair of patterns has associated with it a Jaccard distance, it is possible to think of each statement as a vertex in a simplicial complex. Each vertex is a point, in our case representing a pattern with its descriptors and list of matching subjects. Given a threshold distance, line segments connecting these vertices may be drawn if the Jaccard distance is less than the threshold distance. Those line segments may connect to close triangles, which then may be filled. If all the surfaces of adjacent triangles form a tetrahedron, then the volume may be filled. This can be extended to higher dimensions. Connected segments, areas, and volumes form chains. The set of complexes of dimension *n* is called *C*_*n*_. In this context, for a given threshold, the redescription clusters become nerves. There is a map *∂*_*n*+1_ that extracts the boundaries of the areas in *C*_*n*+1_ and identifies those boundaries in *C*_*n*_. The list *C*_∗_ of all the chains *C*_*n*_ of dimension *n* is called a simplicial complex. It is possible for closed surfaces to enclose a region, yet for there to be no verticies within that region allowing for the surface to be filled. The sets of surfaces enclosing empty volumes are *H*_*n*_=ker(*∂*_*n*_)/Im(*∂*_*n*+1_) form the homology of the simplicial complex, and (loosely) represents the hollow patterns.

The Jaccard distances become the basis of a “filtration” given a set of distance thresholds at which the *C*_*n*_’s and *H*_*n*_’s are evaluated. Shapes, unfilled volumes, etc that persist over some range of thresholds may reveal information about the stability of the relationships among redescription structures and their associated underlying pathological processes, yielding a finer-grained view of the structure of phenotype spaces.

Therefore, we start by computing patterns, and associate these compound patterns with phenotypes possibly marking specific processes. We compute fuzzy redescription clusters in terms of a connectivity associated with a threshold applied Jaccard distances between pattern enrollees. These redescriptions reveal relationships among patterns that may describe disease processes and pathways at specific times of development. Further, information about their topological relationships derived from persistent homology computations also reveal substructures within redescription sets (nerves). All of these pattern relationships were used to characterize phenotypes that used in logistic regressions using glm from R [15] applied to SNPs derived from RAAS complex, and to GWAS logistic regressions performed using PLINK 2.0 [12] to identify genetics related to these patterns.

We sought to test whether the compound phenotype patterns resolved evidence of pathway mechanisms more clearly than simple phenotypes. To achieve this, we applied the RAAS SNPs as described, contrasting results for compound phenotypes were more specific or less specific than the simple phenotypes for three groups of phenotypes, namely type II diabetes, hypertension, and hyperlipidemia. Second, we applied a GWAS to the complex phenotypes in the standard one-test method, and contrasted that to the list of SNPs identified through the combinatorial multi-test method.

## Abbreviations

CAD: Coronary artery disease
GWAS: Genome Wide Association Study
SNP: Single Nucleotide Polymorphism
T2D: Type II Diabetes Mellitus
HTN: Hypertension
DxHT: Diagnosis of Hypertension
DxT2D: Diagnosis of Type II Diabetes Mellitus
DxHL: Diagnosis of Hyperlipidemia
DxCAD: Diagnosis of CAD (>50% occlusion in angiograpy)
Age60: Age 60 or over
SexF: Sex is Female
